# Serum levels of erythropoietin in patients with chronic obstructive pulmonary disease and anemia

**DOI:** 10.1038/s41598-023-34290-w

**Published:** 2023-04-28

**Authors:** Alireza Rezvani, Seyed Masoom Masoompour, Negar Azarpira, Raha Monjazeb, Majid Akbarzadeh, Maryam Salimi, Reza Shahriarirad

**Affiliations:** 1 Department of Internal Medicine, Shiraz University of Medical Sciences, Shiraz, Iran; 2 Thoracic and Vascular Surgery Research Center, Shiraz University of Medical Science, Shiraz, Iran; 3 Non-communicable Diseases Research Center, Shiraz University of Medical Sciences, Shiraz, Iran; 4 Shiraz Transplant Research Center, Shiraz University of Medical Sciences, Shiraz, Iran; 5 Department of Pathology, Shiraz University of Medical Sciences, Shiraz, Iran; 6 Department of Internal Medicine, Lar University of Medical Sciences, Lar, Iran; 7 Student Research Committee, Shiraz University of Medical Sciences, Shiraz, Iran

**Keywords:** Respiration, Haematological diseases, Respiratory tract diseases

## Abstract

The important association of erythropoietin (EPO) serum levels and chronic obstructive pulmonary disease (COPD) with anemia has been inadequately studied and remains a controversial issue. We aimed to shed light on this matter by comparing EPO levels in anemic and non-anemic COPD patients, along with a review of published literature. This cross-sectional study was conducted on COPD patients referred to the pulmonary clinic of Shahid Faghihi Hospital and Motahari clinic, Shiraz, Iran, for one year. We measured complete blood count, red blood cell indices, serum iron, TIBC and ferritin levels, serum EPO levels, and body mass index. Among 35 patients in this study, 28 males and 7 females were enrolled with a mean age of 54.57 ± 8.07 years. The average Forced expiratory volume in first second (FEV1) was 37.26 ± 7.33% and FEV1/FVC was 0.46 ± 0.12. Mean EPO levels were 30.29 ± 2.066 mU/mL. No statistically significant association was observed among erythropoietin levels and Hb, COPD severity, and age. There was no significant difference in EPO levels between anemic and non-anemic patients. EPO level, against the traditional expectation, didn’t increase in COPD patients. EPO production also didn’t compensate for the anemia of chronic disease which considers as a common comorbid disorder in these patients.

## Introduction

Chronic obstructive pulmonary disease (COPD) is a major cause of chronic morbidity and mortality worldwide, characterized by severe irreversible airflow limitation and reduced functional capacity^1,2^. Recent studies show that COPD has the highest proportion of mortality among all respiratory diseases, which accounts for the third leading cause of death in Europe, and the fourth in the United States^3,4^. The prevalence of COPD was reported as 5.57% in a systematic review and meta-analysis in Iran^5^. The severity of COPD can be influenced by various internal and external factors^6,7^. Furthermore, COPD is associated with extrapulmonary effects and markers of systemic inflammation^8^. These extrapulmonary manifestations and co-morbidities are either secondary to the inflammatory burden of COPD or occurring in association with COPD due to sharing of the same risk factors^9^. Cigarette smoking is the main contributor of COPD. Dyspnea at rest appears when COPD worsens, and doing daily activities becomes more difficult^10^. Opium is beneficial in treating dyspnea, according to evidence from multiple studies; However, the effects of opium on lung volumes have not been examined^11–13^.

Anemia is a well-known manifestation of chronic illness, could be present among patients with COPD^14–16^. Multiple various factors may contribute to the occurrence of anemia in COPD, including iron and vitamin deficiency, anemia of chronic disease related to inflammation, hypogonadism, comorbidities, or treatment related^17^. Several studies have reported a prevalence of anemia ranging from 7.5 to 21% in COPD populations of varying severity^14–16^. The clinical impact of anemia is significant; it has been associated with increased dyspnea, reduced exercise capacity, higher costs of care, and increased morbidity in COPD patients^18,19^, Nevertheless, often the symptoms of anemia are not apparent in COPD patients^14,20^.

Although the exact origin of anemia in COPD patients is uncertain, there seems to be a correlation with several pro-inflammatory indicators, indicating that at least a portion of the anemia—i.e., the anemia of chronic inflammation—is caused by inflammation^10^. In spite of the association between anemia and COPD, the role of erythropoietin (EPO) in this content is still unclear. EPO promotes the production of red blood cells and applies its hematopoietic properties through stimulating the proliferation of early erythroid precursors and the differentiation of later precursors of the erythroid lineage^21^. The major stimulus for EPO production and the increase is diminished arterial oxygen content associated with anemia or hypoxia^22,23^.

To replace those lost to senescence, healthy human adults produce about 200 billion red blood cells (RBCs) each day. An exquisitely regulated oxygen-sensing mechanism, known as erythropoiesis, has evolved to maintain RBC counts within a specific physiological range^24–26^. EPO, a cytokine secreted by the kidney in response to low blood oxygen tension, is essential to this mechanism. A number of signaling pathways are activated when circulating EPO binds to its cognate receptor (EPOR) on bone marrow erythroid progenitors, supporting differentiation into mature RBCs. Numerous human diseases linked to excess or insufficient RBC production are caused by inherited and acquired abnormalities in EPO production, its downstream activities, or its regulation^27^.

Therefore, in this study, we aim to shed a light on erythropoietin serum levels in patients with chronic obstructive pulmonary disease (COPD) and anemia, along with a review of previously reported studies.

## Material and method

### Study population

This study is a cross-sectional study on COPD patients referring to the pulmonary clinic of Shahid Faghihi Hospital and Motahari clinic, Shiraz, Iran, during a one-year period (October 2016–2017). Based on statistical calculation, a sample size of 35 patients was selected (*P* = 27%, P0 = 50%, a2 = 0.05, b = 0.2, power = 80%). Patients data including their age, gender, comorbid disease, and opium use were documented. The parameters measured in each patient included complete blood count (CBC), red blood cell indices, serum iron, total iron-binding capacity (TIBC) and ferritin levels, serum EPO levels, and body mass index (BMI). Cell blood counts and red blood cell indices were determined by an automated H analyzer. Serum EPO levels were determined by the ELISA method with an R& D kit on a venous blood sample collected in the morning at 8 A.M. Normal EPO levels based on our kit was considered between 4 and 24 mU/ml. Blood and serum samples were obtained from the participants for laboratory evaluation of the levels of B12, folic acid, along with liver and renal function tests.

COPD diagnosis was based on the guidelines of the American Thoracic Society^28^, i.e. Forced expiratory volume in first second (FEV1) < 80% of predicted and FEV1/ Forced vital capacity (FVC) < 0.7 with FEV1 change of fewer than 200 ml and 12% in the post-bronchodilator test. The severity of COPD was assessed using FEV1 measures based on the global initiative for chronic obstructive lung disease (GOLD) guideline^29^. All patients received routine standard therapy for COPD, individually. Anemia diagnosis was based on hemoglobin levels and classified based on WHO guidelines, and included under 12 mg/dl for female and under 13 mg/dl for male patients^30^.

The inclusion criteria for our study regarded patients aged above 40 years old, with a definite diagnosis of COPD and anemia. Exclusion criteria included exacerbation of COPD, asthma(defined as an increase in FEV1 more than 12% and 200 ml above baseline after administration of a short-acting bronchodilator), recent blood transfusion, patients with cancer, severe liver or kidney diseases, left heart failure, or other chronic diseases, history of gastrointestinal bleeding or blood loss of any other cause, vitamin B12 or folic acid deficiency (microcytic and macrocytic anemia), low ferritin serum levels (less than 100 ng/ml), and positive drug history for ferrous sulfate, folic acid, and vitamin B12.

### Statistical analysis

The data were analyzed by using SPSS software version 22. Normality tests were performed. Comparison of quantitative variables between anemic and nonanemic groups was performed by independent t-test and Mann–Whitney test, accordingly. For analysis of the association between hemoglobin and EPO, the Spearman correlation coefficient was used. Association of patients factors, including demographical factors (age, gender), and also opium use was also evaluated with spirometry results and anemia. For analysis of qualitative variables, we utilized the Chi-square test. *P*-values less than 0.05 were considered significant.

### Ethics approval and consent to participate

The ethics committee of Shiraz University of Medical Sciences approved this study. Patients' information was de-identified before data analysis and confidentiality of patient information was guaranteed and protected. Written inform consent was obtained from all participants prior to evaluation. The study was conducted in compliance in accordance with the relevant guidelines and regulations and the Declaration of Helsinki.

## Results

The 35 patients in this study included 28 male and 7 female patients with a mean age of 54.57 ± 8.07 years. The demographic and clinical features of the patients are demonstrated in Table 1.

**Table 1:** Baseline demographic and clinical features of COPD patients.

Variable	Mean ± SD or Median [IQR]	Reference range
Age (years)	54.57 ± 8.07	–
Spirometry finding		
FEV1(% of predicted)	37.26 ± 7.34	≥ 80%
FEV1/FVC	46.57 ± 12.16	Within 5% of the predicted ratio
Lab data		
Erythropoietin (mU/mL)	30.29 [28–32]	4–26
Hemoglobin (g/dl)	10 [8–12]	Male ≥ 13; Female ≥ 12
Aspartate aminotransferase (U/L)	25 [15–30]	10–40
Alanine aminotransferase (U/L)	24 [18–29]	7–56
Alkaline phosphatase level test (u/l)	211 [131–249]	44–147
Direct Bilirubin (mg/dl)	0.2 [0.1–0.3]	0.3
Total Bilirubin (mg/dl)	0.6 [0.1 – 0.3]	1.2
Blood urea nitrogen (mg/dl)	14﻿ [11–16]	7—20
Creatinine (mg/dl)	0.7 [0.6–1.00]	Male: 0.74 – 1.35; Female: 0.59 – 1.04
Vitamin B12 (pg/ml)	512 [363–649]	160—950
Folic acid (ng/ml)	13 [9–16]	2.7—17.0
Ferritin (ng/ml)	146 [92–208]	20—250
Serum Iron (mcg/dl)	101 [78–113]	60—170
Total iron binding capacity (mcg/dl)	339 [288–380]	240—450
White blood cell count (count/ml)	6 [5–9]	4.5–11
Platelet count (count/ml)	268 [206–384]	150–450
Mean corpuscular volume (fl)	87 [82–92]	80–100
Mean corpuscular hemoglobin. (pg)	31 [27–33]	27.5–33.2
Red cell distribution width (%)	13 [12–14]	12.2–16.1

As seen in Table 1, the average FEV1 and FEV1/FVC was 37.26 ± 7.33% and 0.46 ± 0.12, respectively. Based on the grading of COPD severity, none of our patients had mild or moderate severity. Also, opium use had a significant correlation with FEV1 (Pearson correlation = 0.356, *P* = 0.036). The association between the patients’ features and anemia is demonstrated in Table 2.

**Table 2:** Evaluation anemia among clinical features of COPD patients.

Variable	Overall	Anemia		*P*. value
		Yes (*n* = *25)*	No (*n* = *10)*	
Age; mean (SD)	54.57 (8.07)	53.16 (7.9)	58 (7.78)	0.103
Male gender; n (%)	28 (80)	20 (80)	8 (80)	1.000
Erythropoietin; mean (SD)	30.29 (2.07)	30.44 (1.98)	29.9 (2.33)	0.493
FEV1; mean (SD)	37.26 (7.33)	36.28 (7.42)	39.7 (6.88)	0.218
FEV1/FVC; mean (SD)	46.57 (12.16)	45.72 (11.81)	48.7 (13.4)	0.521
COPD Severity; n (%)				0.644
Severe	28 (80)	19 (76)	9 (90)	
Very severe	7 (20)	6 (24)	1 (10)	
EF; mean (SD)	62.97 (4.73)	63.48 (4.75)	61.7 (4.67)	0.322
Systolic blood pressure; mean (SD)	14.66 (3.24)	14.72 (3.34)	14.5 (3.17)	0.859
Opium; n (%)	24	14(56)	10(100)	0.015

*SD* standard deviation, *FEV1* forced expiratory volume in one second, *FVS* forced vital capacity, *COPD* chronic obstructive pulmonary disease, *EF* ejection fraction.

In our study, we focused on the erythropoietin levels and their correlation with factors such as Hb and COPD severity. As demonstrated in Table 3, although, a positive correlation, no statistically significant association was observed among erythropoietin levels and Hb, COPD severity, and age.

**Table 3:** Correlation among erythropoietin levels and COPD patients features.

Variable	Erythropoietin Level	
	*P*. value*	Coefficient correlation
Hb Level	0.843	0.035
COPD Severity	0.606	0.090
Age	0.634	0.083
FEV1	0.856	0.032
FEV1/FVC	0.940	0.013

*Pearson Correlation for age, Hb levels, FEV1 and FEV1/FVC ratio; Spearman correlation for COPD severity.

## Discussion

Although a higher level of EPO followed by hypoxia had been traditionally expected in COPD patients’ further studies frequently report a normal or lower level of EPO in comparison to their healthy counterparts^20^. Despite many studies, controversy continues regarding this issue and its possible pathophysiology. In this perusal, in addition to measuring the serum level of EPO in COPD patients and assessing its relation with other factors such as HB level, we tried to review previous studies to get a comprehensive view about this issue and its various aspects, as well as the possible reasons.

The cumulative evidence in our study indicates that EPO level didn’t increase in COPD patients even in very severe stage and also there was no significant correlation between the mentioned level and disease severity. Contrary to our results, many researchers reported the higher-than-normal level of EPO in COPD patients and also explained their result with respect to hypoxia caused by chronic airway obstruction along with anemia of chronic disease^31–33^ In this regard, Sharma et al. conducted a study in 2015 on 200 COPD patients and reported a significant rise in EPO level with increasing the severity of the disease^34^. On the other hand, other studies provided evidence in favor of lower production of EPO. They attributed the dull endogenous EPO response to hypoxia to cytokines and inflammatory factors which commonly increase in COPD patients. In other words, normal increases in the level of EPO at the presence of hypoxia were not achieved in COPD conditions. Some of them also measured the EPO level in severe and critically ill respiratory or non-respiratory related patients and concluded that EPO response to hypoxia decrease in presence of inflammation^35,36^. Sala et al.^37^ and El Gazzar et al.^38^ assessed almost 100 COPD patients in total and both of them reported the lower level of serum EPO in the acute inflammatory phase of COPD exacerbation.

In continuation of the discussion, some studies evaluated the EPO serum level in different stages of COPD. They unanimously came to this conclusion that although the average level of EPO level is lower in COPD patients, it differed in various stages of the disease which the highest level measured in stages 2 and 3 of the disease and the mentioned serum level was low at the first and last stage^38,39^. The lower level of EPO in the first stage came back to the near-normal saturation level and so lack of enough hypoxia to stimulate the EPO production, and about the last stage, it may be related to the excess inflammatory factors which prevent the expected EPO production in presence of considerable hypoxia.

From the point of production origin of EPO view, kidney mostly, there are studies which reported chronic renal failure in COPD patients even with normal kidney-related functional serum markers (e.g., blood urea nitrogen and creatinine) following the impairment of EPO production^40,41^. Incalzi et al. pointed to the kidney-related adverse effects of drugs which commonly used in COPD management (e.g., hydrosoluble drugs and antibiotic which used in COPD exacerbation) or drugs that frequently used to treat comorbid conditions (e.ge. digoxin for atrial fibrillation or thiazides for hypertension)^41^. It should be taken into consideration that several common simultaneous disorders such as diabetes mellitus or hypertension, not only due to adverse reactions of related drugs but also because of the own pathophysiology impact, can impair the function of the kidney as a main production source of EPO. However, focused studies in this regard are justified.

Notwithstanding the foregoing, EPO level can be expected logically lower in COPD patients particularly in exacerbation periods and end stages of the disease. Without any doubt, the prospective cohort studies with a large sample of COPD patients in various stages can assess the associated factors in blunted EPO response to hypoxia for further decision in management. Furthermore, molecular and tissue studies related to EPO production can help significantly to find out the exact pathophysiology of the mentioned contradictory response of EPO to hypoxia in COPD patients.

Based on the result of the current study no statically significant association was found between the EPO serum level and Hb level in COPD patients. In this regard, Markoulaki et al. found a substantial positive correlation. However, their study was limited by not evaluating the patients' iron profile-related data to exclude the anemia and low HB level followed by iron deficiency. An almost similar bias was found in the study conducted by Attaran et al. who reported that there is no significant correction between HB level and EPO level in anemic COPD patients, while their study didn’t measure the level of vitamin B12 and folate to exclude the combined anemia and so their results attributed the total anemic patients and low HB level to COPD conditions^20^. On the contrary, several studies reported a significant inverse correlation between HB level and EPO level. In other words, the higher the EPO level, the lower the HB level. They explained that normal bone marrow response to EPO rising as well as iron transportation is impaired in COPD patients^31,33,34,42^. Therefore, elevated serum EPO couldn’t result in increasing HB level which occurred due to chronic disease. Overall, it seems that EPO can’t compensate completely for the anemia and low HB level in COPD patients.

Our study has several limitations. The sample size is small and mild to moderate COPD didn't find in our sample so we can't evaluate the EPO level according to different stages of the disease. Another limitation was that we didn't consider the past drug history of patients such as corticosteroids, antibiotics, or any other drugs which have potential renal toxicity effect or influence the inflammatory response. Furthermore, we didn't evaluate recent oxygen therapy management which can affect the EPO levels. Our study was also conducted among hospitalized patients, and patients with lower severity of COPD who didn’t require hospitalization were not included. Further studies while considering these factors, along with a control group including the non-COPD patients are necessary to increase our understanding of EPO among COPD patients.

## Conclusion

EPO level, against the traditional expectation, didn't increase in COPD patients. EPO production also didn't compensate for the anemia of chronic disease which considers as a common comorbid disorder in these patients.

## Data Availability

SPSS data of the participant can be requested from the corresponding author. Please write to the corresponding author if you are interested in such data.
